# The Role of Exclusive Autologous Lipotransfer in Non-Irradiated Breasts After Mastectomy

**DOI:** 10.3390/jcm14134468

**Published:** 2025-06-24

**Authors:** Aikaterini-Gavriela Giannakaki, Eftychia Papachatzopoulou, Ioannis Papapanagiotou, Sophia Koura, Dimitris Baroutis, Spyridon Marinopoulos, George Daskalakis, Constantine Dimitrakakis

**Affiliations:** First Department of Obstetrics and Gynecology, Alexandra University Hospital, National and Kapodistrian University of Athens, 11528 Athens, Greece; efipapaxatzopoulou@hotmail.com (E.P.); gpapamd@hotmail.com (I.P.); sophia_koura@hotmail.com (S.K.); dbaroutis@gmail.com (D.B.); smarinopoulos@outlook.com (S.M.); gdaskalakis@yahoo.com (G.D.); dimitrac@ymail.com (C.D.)

**Keywords:** autologous fat graft, adipose-derived stem cells (ADSCs), implant-based reconstruction (IBR), patient satisfaction, complication rates, oncological safety, artificial intelligence (AI)

## Abstract

**Background/Objectives:** Autologous fat grafting (AFT) has become a widely used technique in breast reconstruction, offering natural aesthetics, tissue integration, and patient satisfaction. However, its clinical outcomes require comparison with implant-based reconstruction (IBR), the most common method in clinical practice. While AFT provides a more natural appearance and avoids foreign body-related complications, issues such as fat resorption, procedural variability, and oncological concerns necessitate further investigation. Additionally, artificial intelligence (AI) has been increasingly integrated into breast imaging and reconstructive planning, improving diagnostic accuracy, procedural optimization, and complication prevention. This study aims to compare AFT and IBR while exploring AI’s role in enhancing breast reconstruction outcomes. **Methods:** A comprehensive review of clinical studies was conducted to evaluate the advantages, limitations, and oncological implications of AFT versus IBR. AI-driven applications in breast imaging and reconstructive planning were examined for their potential in predicting fat graft retention and optimizing implant selection. Data from systematic reviews and meta-analyses were incorporated to refine reconstruction strategies. **Results:** AFT offers superior aesthetic outcomes with better tissue integration but presents variability in fat resorption. IBR remains the preferred approach due to its predictability but carries risks of implant-related complications. AI technologies contribute to improved reconstruction planning, enhancing surgical precision and long-term patient outcomes. **Conclusions:** Optimized patient selection and long-term follow-up are essential for improving breast reconstruction techniques. AI-driven approaches provide valuable tools for enhancing procedural predictability and personalized treatment strategies. Future research should focus on refining AI algorithms and establishing standardized protocols for reconstructive decision-making.

## 1. Introduction

Breast reconstruction has undergone significant advancements, with autologous fat transfer (AFT) emerging as both a complementary and standalone technique. While initially utilized to correct contour irregularities, refinements in surgical methods have enabled AFT to serve as a primary reconstructive modality, providing a natural breast appearance and texture [1].

Unlike implant-based or flap-based techniques, AFT utilizes the patient’s own adipose tissue, harvested via liposuction, to restore breast volume while minimizing the risk of foreign body reactions. Various liposuction and fat transfer techniques—including active filtration, low-pressure decantation, and standard decantation—affect fat viability and tissue integration differently, necessitating careful selection to optimize outcomes [2].

Recent advancements in adipose-derived regenerative medicine have introduced the concept of stromal-vascular fraction (SVF) enhancement to improve graft survival. SVF comprises adipose-derived mesenchymal stromal cells (ASCs), endothelial cells (ECs), endothelial progenitor cells (EPCs), pericytes, preadipocytes, and hematopoietic cells, all of which contribute to vascularization and functional adipose tissue formation in the recipient area. These cells seem to offer higher regenerative potential due to their ability to differentiate into various cell types and promote angiogenesis [3,4].

Compared to implant-based reconstruction, AFT eliminates foreign materials, promoting superior tissue integration while reducing long-term complications. Moreover, it offers high patient satisfaction rates, yielding aesthetically natural results with minimal morbidity. Nonetheless, AFT demands precise surgical execution, including optimal reinjection volume and scar tissue management, to ensure durability and efficacy [1,2].

## 2. Materials and Methods

### 2.1. Study Design

This comprehensive review was conducted to synthesize existing literature on the role of exclusive autologous lipotransfer in breast reconstruction after mastectomy and compare it with implant-based reconstruction. Although a structured and systematic approach was applied, this review does not adhere to PRISMA guidelines, as it is not a systematic review or meta-analysis.

### 2.2. Search Strategy

A comprehensive literature search was conducted using the PubMed database to identify studies published between 2020 and 2025 that evaluate exclusive autologous lipotransfer in breast reconstruction. To ensure an optimized, thorough, and structured search, the literature search used the following MeSH terms: ‘Lipotransfer’, ‘Autologous Fat Graft’, ‘Fat Grafting’, ‘Lipofilling’, combined with ‘Mastectomy’ and ‘Breast Reconstruction’. All relevant articles fitting the inclusion criteria were incorporated.

### 2.3. Inclusion and Exclusion Criteria

Inclusion Criteria

- Prospective studies
- Retrospective studies
- Randomized clinical trials (RCTs)
- Systematic reviews and meta-analyses
- Women post-mastectomy

Exclusion Criteria

- Case reports
- Duplicate publications
- Non-relevant articles
- Women after breast-conserving surgery
- Patients with irradiated breasts

### 2.4. Study Selection

The selection process was conducted by two independent researchers. Non-relevant articles were excluded based on title and abstract review. Full-text articles were thoroughly evaluated, and studies focusing on exclusive autologous lipotransfer reconstruction post-mastectomy were included in the final analysis.

Patients who had undergone radiation therapy were excluded from this review to ensure uniformity in the analysis and avoid potential confounding factors. The goal of this study is to evaluate autologous fat transfer in non-irradiated patients as a primary reconstructive option, focusing on fat graft survival and tissue integration without the influence of radiation-induced changes. While the role of fat grafting post-radiotherapy is a significant topic in reconstructive surgery, it falls beyond the scope of this review and should be further explored in future studies.

To enhance the depth of the review, the authors combined data from selected studies, reinforcing the existing literature with conclusions on effectiveness, safety, complication rates, patient satisfaction, and comparisons between implant-based and autologous lipotransfer reconstruction.

Minor language editing support was provided using Microsoft Copilot (2024 version). All content was critically reviewed and finalized by the authors.

## 3. Results

### 3.1. Techniques for Fat Graft Injection

Optimizing fat graft injection methods is essential for enhancing long-term volume retention in breast reconstruction. Various processing techniques impact fat viability, tissue integration, and overall graft success.

Fat Processing Techniques and Retention Rates.

Current fat grafting techniques include:

- Active filtration
- Low-pressure decantation
- Standard decantation, each influencing cell survival and inflammation differently.

Studies indicate that both active filtration and low-pressure decantation yield higher fat retention rates compared to traditional decantation (*p* < 0.05). These methods preserve viable adipocytes, reducing tissue resorption and necrosis while minimizing inflammatory cellular debris.

#### Centrifugation-Based Fat Processing

Traditional autologous fat grafting is widely used in plastic and reconstructive surgery, but its effectiveness is often limited by unpredictable fat retention, with resorption rates ranging from 30% to 70% within the first year [5]. Fat tissue contains a significant number of stem cells, second only to bone marrow, and harvesting and enhancing stem cells could improve the procedure’s effectiveness. These cells play a crucial role in vascularization and tissue regeneration, improving graft integration [6]. Cell-assisted lipotransfer (CAL) has been explored for oncologic reconstruction following radiation, facial rejuvenation, and breast augmentation, showing promising results in improving fat graft retention and tissue remodeling. Several techniques have been explored. Specifically, stromal vascular fraction (SVF) is a heterogeneous cell population derived from adipose tissue that contains endothelial progenitor cells, fibroblasts, mesenchymal stem cells, and immune cells. Its regenerative properties support vascularization, improving graft survival and tissue integration [7]. Also, platelet-rich plasma (PRP) is an autologous blood-derived product rich in growth factors, including platelet-derived growth factor (PDGF) and vascular endothelial growth factor (VEGF). These factors promote angiogenesis, enhancing fat retention and tissue remodeling [7]. Another technique is the adipose-derived stem cell (ADSC) isolation, and these cells are abundant in adipose tissue and play a key role in cellular regeneration and vascular support [5]. Research by Schuchun Hu et al. found that centrifugation-based fat processing resulted in a higher retention rate (51.5%, 95% CI: 41.5–61.5%) compared to sedimentation (38.7%, 95% CI: 30.9–46.5%). Stromal vascular fraction (SVF) enrichment achieved via centrifugation improved fat survival (weighted mean difference: 17.36, 95% CI: 8.84–25.87) [7]. While centrifugation appears to enhance long-term retention, further studies are necessary to verify its efficacy and standardize optimal processing protocols.

### 3.2. Patients’ Satisfaction

Satisfaction rates with autologous fat transfer (AFT) were notably high, particularly in relation to breast ptosis correction and overall aesthetic outcomes [8]. Studies have shown that sexual well-being scores were significantly higher among AFT patients compared to those who underwent implant-based reconstruction [9].

Additionally, liposuction did not restrict future corrective procedures, enabling patients to make adjustments as needed, further supporting AFT’s viability as a reconstructive option for women seeking moderate breast reconstruction with minimal recovery time.

In a randomized clinical trial, patients who underwent exclusive AFT for total breast reconstruction post-mastectomy reported higher quality-of-life outcomes [10]. However, 6 out of 91 patients in the AFT group discontinued treatment early, citing the procedure as too demanding, exhausting, or difficult to continue [10].

### 3.3. Complication Rate

Autologous fat transfer (AFT) is widely regarded as a safe and effective breast reconstruction technique, though like all surgical procedures, it is not without potential complications.

A randomized clinical trial involving 16 patients examined the effects of Botulinum Toxin A (BT-A) in combination with AFT, revealing that BT-A improved fat graft retention compared to saline-assisted lipofilling [11]. Fat resorption is a major limitation in breast lipofilling, with studies reporting 40–60% absorption within the first 6 months [12,13]. As a result, patients often require multiple lipofilling sessions to achieve the desired breast volume. In the study of Piffer et al., the average number of sessions was 2.2, with patients undergoing lipofilling over a period of 6.8 months. However, those with larger breast sizes (C or D cup) needed more than 3 sessions, likely due to higher fat resorption rates [13]. Another study concluded that CAL and PRP-assisted lipotransfer significantly improved the fat survival rate (CAL vs. non-CAL: 71% vs. 48%, *p* < 0.0001; PRP vs. non-PRP: 70% vs. 40%, *p* < 0.0001; CAL vs. PRP: 71% vs. 70%, *p* = 0.7175) [14].

A bicentric retrospective observational study reinforced the growing acceptance of exclusive lipotransfer, confirming its low complication rate. The most reported postoperative complications included fat necrosis cysts (11%), infections (4.4%), and hematomas (2.2%) [15]. Fat necrosis in the breast is a benign inflammatory condition resulting from aseptic fat saponification. Its clinical and imaging presentation can mimic malignancy, leading to unnecessary concern and additional diagnostic procedures. The presence of irregular masses, skin retraction, or nodular formations often necessitates careful differentiation through mammography, ultrasound, and MRI, though biopsy remains the definitive diagnostic method when imaging findings are inconclusive. Fat necrosis may contribute to aesthetic concerns, particularly when calcifications develop, requiring corrective intervention. Patients undergoing autologous fat transfer often undergo routine surveillance due to oncofobia, emphasizing the need for precise imaging interpretation to prevent unwarranted biopsies [16].

Another study found that seroma, hematoma, and infection were relatively infrequent following AFT [17].

Further research is necessary to assess the long-term effects of donor site complications [18].

#### Safety Profile of AFT

Despite these risks, serious adverse effects remain rare. A randomized clinical trial evaluating the safety of breast lipotransfer found no cases of fat embolism, vascular injury, nervous injury, infection, or prolonged bruising [19]. This suggests that with appropriate techniques, AFT maintains a favorable safety profile.

### 3.4. Oncological Safety

The oncological safety of autologous fat transfer (AFT) in breast reconstruction remains a critical area of study. While findings indicate that 29 out of 583 non-irradiated mastectomy patients experienced recurrence (*p* = 0.014), AFT appears oncologically safe, though further research is necessary to establish definitive safety guidelines, particularly for irradiated patients [20]. Basic science studies suggest that adipose-derived stem cells (ADSCs) may promote breast cancer growth, but fat grafting alone does not [21].

#### 3.4.1. AFT and Recurrence Risk

Several studies affirm that AFT does not increase the risk of loco-regional recurrence or distant metastasis [20,21,22].

However, caution is advised when using adipose-derived stem cells (ASCs), as experimental data suggests a potential link between ASCs and cancer progression [21]. Gentile et al. also confirmed that AFT was not associated with increased loco-regional recurrence, reinforcing its safety for breast reconstruction following cancer treatment [23].

#### 3.4.2. Comparative Outcomes Between AFT and Control Groups

There was no significant difference in overall disease-free survival and local recurrence rates between AFT patients and control groups [24]. A multicenter study reported local recurrence rates of 3.9% in the AFT group and 6.1% in the non-AFT group, suggesting non-inferiority of AFT (*p* = 0.084). Across different biological subtypes, recurrence patterns remained consistent, with the following adjusted hazard ratios (aHRs): Luminal-like group: aHR = 0.76, *p* = 0.493, HER2-enriched group: aHR = 0.89, *p* = 0.882, TNBC group: aHR = 0.61, *p* = 0.543 [25].

### 3.5. Comparison Between Exclusive Autologous Fat Grafting and Implant-Based Reconstruction

A multicenter prospective study comparing implant-based breast reconstruction (IBR) with exclusive autologous fat transfer (AFT) after mastectomy found that AFT patients experienced a mean graft survival rate of 37.1%, indicating that approximately one-third of transferred fat remained viable 12 months postoperatively [18]. Patients who underwent implant-based reconstruction achieved significantly higher postoperative breast volumes, with an 83.8 mL difference favoring implants (*p* < 0.001). However, fat graft survival stabilized between 6 and 12 months, suggesting that breast volume is retained beyond six months. Factors influencing graft survival include patient population, fat preparation method, volume measurement techniques, number of fat grafting sessions, and injected fat grafting volume [7]. While IBR offers superior volume retention, AFT provides a more natural aesthetic and greater flexibility [26,27]. Nonetheless, plastic surgeons remain cautious about transferring excessive fat in a single session, as overloading may hinder graft viability. Despite these concerns, exclusive AFT after mastectomy has demonstrated effectiveness, yielding higher patient and plastic surgeon satisfaction across multiple domains [10,15].

#### 3.5.1. Economic and Quality-of-Life Comparisons

A randomized Dutch clinical trial comparing AFT and IBR revealed that while AFT was initially more expensive, it became cost-effective over a ten-year period and remained significantly more economical over thirty years [15]. Quality-of-life outcomes further reinforced AFT’s advantages, with EQ-5D-5L QALY scores proving superior in AFT patients (0.83) versus IBR patients (0.79). Additionally, studies reported higher satisfaction levels among AFT patients compared to implant recipients. Specifically, higher BREAST-Q scores were observed in the AFT group, with significant improvements noted in satisfaction with breasts, physical well-being, and overall satisfaction with outcomes [10].

Quality of life improved over time more in the AFT group than in the IBR group [10]. However, according to complication rates, 18 patients in the IBR group discontinued treatment due to implant aversion, while six patients in the AFT group stopped due to treatment burden [10].

#### 3.5.2. Aesthetic and Safety Considerations

On the other hand, Wederfoort et al. found that aesthetic outcomes varied significantly, underscoring the need for personalized patient counseling before selecting a reconstructive approach. Autologous fat transfer offers a minimally invasive technique with potentially natural results, but aesthetic evaluation remains subjective, necessitating clear patient expectations before treatment [18]. A multicenter study observed that AFT carries higher complication rates than breast implants: major complications: 3.2% in AFT vs. 2.3% in implants, infection risk: 1.1% in AFT vs. 0.5% in implants [27]. Although fat grafting is a viable alternative to implants, it presents higher infection risks, particularly in smokers and high-risk patients, as well as higher absorption rates [27]. While AFT is widely utilized in breast reconstruction, its long-term effects on quality of life compared to prosthetic reconstruction remain inconclusive, necessitating further high-quality studies to establish solid evidence [28] (Table 1).

### 3.6. Hybrid Reconstruction

In clinical practice, autologous fat transfer is frequently combined with implant-based breast reconstruction to optimize aesthetic outcomes. Hybrid breast reconstruction enhances contour and softness and reduces implant-related complications. Studies indicate that the mean number of fat grafting sessions per breast ranges from 1.3 to 3.2, with an average volume of 59 to 313 mL per session, supporting its effectiveness in contour enhancement. The complication rate in hybrid reconstruction is 7.9%, comparable to standard implant-based reconstruction, but may offer long-term advantages in reducing capsular contracture risks. Patients report high satisfaction, with hybrid reconstruction improving symmetry and natural feel, making it a preferred option in selected cases [29].

Second-stage fat grafting (during the exchange of tissue expander for a permanent implant) provides similar or superior clinical outcomes compared to third-stage (after implant placement, often months later) fat grafting (*p* = 0.178). BREAST-Q scores show no significant difference in patient satisfaction between the two approaches. Second-stage AFT does not increase revision surgery rates, suggesting it may be a more efficient option in IBR [30].

## 4. Discussion

Breast reconstruction is essential for post-mastectomy recovery, offering plenty of surgical options to restore breast shape and enhance quality of life. Autologous fat grafting (AFT) has evolved in the last years as a viable option for breast reconstruction, as an additional tool in the traditional methods of breast reconstruction (implants and autologous tissue) or as an exclusive method after mastectomy or breast-conserving surgery. The role of exclusive lipofilling after mastectomy has gained ground, offering natural appearance of breast and reporting high satisfaction rates among both patients and surgeons (93%) [31]. Autologous lipotransfer and implant-based reconstruction (IBR) represent two primary approaches, each with distinct advantages and limitations. This discussion analyzes the role of exclusive autologous lipotransfer in non-irradiated breasts after mastectomy, focusing on clinical outcomes, oncological safety, patient satisfaction, and emerging advancements such as artificial intelligence (AI) integration in breast reconstruction.

Comparative Analysis of Fat Graft Injection Techniques in Breast Reconstruction

Several fat processing techniques have been developed to enhance graft viability and long-term volume retention in breast reconstruction, including active filtration, sedimentation, and cellular enrichment strategies such as cell-assisted lipotransfer (CAL). However, the evidence regarding their efficacy remains inconsistent.

Hu et al. [32] concluded that CAL is associated with improved fat retention and volume augmentation, although patient satisfaction was comparable to conventional autologous fat transfer (AFT). Similarly, Ming Li et al. [11] reported significantly higher fat survival in the CAL group compared to standard lipotransfer (SMD = 1.79, 95% CI = 0.28–3.31; *p* = 0.02), whereas SVF-enhanced grafting showed no statistical difference (SMD = 1.52, 95% CI = –0.21–3.24; *p* = 0.08).

Conversely, other studies failed to demonstrate a clear benefit of CAL. A randomized clinical trial found no significant improvement in volume retention with ASC enrichment [33]. Complication rates, including fat necrosis and need for repeat procedures, were comparable across techniques [34].

A broader review by Lutfi et al. [35] described fat retention rates ranging from 39% to 76% for conventional AFT, with CAL offering up to a 24% increase. Nevertheless, the added complexity and cost of ASC-enrichment may not justify its use in all patients, especially those undergoing aesthetic augmentation rather than oncologic reconstruction.

Overall, while cellular enrichment techniques like CAL may offer modest improvements in fat retention, their clinical benefit remains context dependent. Further randomized studies with standardized protocols are needed to determine their routine value in breast reconstruction.

Comparison and Clinical Outcomes Comparative Analysis of AFT vs. IBR

Recent studies highlight the superior aesthetic outcomes associated with AFT, which provides a more natural tissue composition, avoids prosthesis-related complications, and minimizes foreign body responses. Mangialardi et al. [36] demonstrated that AFT offers a viable alternative to implants, yielding natural breast contours and high patient satisfaction with cosmetic results. However, reductions in physical well-being scores were observed, potentially reflecting the demands of postoperative recovery.

One major limitation of AFT is the variable fat resorption rate, with graft survival typically stabilizing within 6 to 12 months. Multiple sessions are often required to achieve optimal volume restoration. Nonetheless, patient-reported outcomes consistently favor AFT in terms of satisfaction with breast appearance compared to IBR.

In contrast, IBR enables greater volume stability and immediate contour restoration, making it a preferred option for patients seeking prompt reconstruction following mastectomy. Despite these advantages, IBR is associated with potential complications such as capsular contracture, implant migration, and foreign body reactions. Psychological factors, including implant aversion, have contributed to higher dropout rates in studies comparing the two modalities.

From a clinical standpoint, the choice between AFT and IBR should be personalized and guided by factors including anatomical suitability, oncologic safety, comorbidities (e.g., diabetes, smoking), psychosocial preferences, and institutional resources.

In non-irradiated patients with adequate donor fat, AFT may be preferred due to its physiological integration, lower rate of prosthetic complications, and more natural aesthetic. However, the unpredictability of fat survival, need for staged procedures, and technical variability require thorough preoperative counseling.

Conversely, IBR may be indicated in patients desiring immediate reconstruction, or when AFT is not suitable due to insufficient donor fat, extensive defects, or lack of access to experienced surgical teams. Although prior radiotherapy was traditionally considered a relative contraindication to AFT, primarily due to concerns about graft viability and increased complication rates, accumulating evidence has since challenged this notion. Recent studies have demonstrated that AFT can be safely and effectively applied in previously irradiated patients, both as a standalone reconstructive method and in conjunction with implants, without a significant increase in complication rates [37,38].

A shared decision-making model is essential, integrating patient expectations with oncologic parameters, surgical expertise, and long-term reconstruction goals.

Oncological Safety of AFT

One of the most critical concerns in clinical practice is the oncological safety of autologous fat transfer (AFT). Our findings, in line with current literature, support that AFT does not increase the risk of local recurrence or distant metastasis in breast cancer survivors, thus reinforcing its safety as a reconstructive option. Nevertheless, the potential role of adipose-derived stem cells in tumorigenesis remains under investigation. While experimental models have demonstrated interactions between ASCs and breast cancer cells, no robust clinical data currently link AFT to increased oncological risk. Continued long-term surveillance and standardization of fat processing techniques may further strengthen its oncological profile.

Clinical Outcomes, Complications, and Patient Satisfaction

AFT demonstrates an acceptable safety profile, with no major complications recorded. Minor events, including ecchymosis, localized pain, and temporary asymmetry, were observed [20]. Notably, fat graft resorption, often ranging between 30–50% within the first postoperative year, remains a limitation, sometimes requiring additional sessions to maintain volume.

When compared to IBR, AFT may carry slightly higher minor complication rates (3.2% vs. 2.3%), with an infection risk of 1.1% compared to 0.5%, particularly among smokers. However, BREAST-Q scores consistently favor AFT in terms of satisfaction with aesthetic results, physical well-being, and psychosocial adjustment. These findings support the growing role of AFT, either as a stand-alone method or adjunctively, in achieving more natural reconstruction outcomes.

The Role of Artificial Intelligence (AΙ) in Breast Reconstruction

Artificial Intelligence (AI) is increasingly transforming the field of breast reconstruction by providing advanced tools that support surgical decision-making, enhance outcome prediction, and personalize patient care. AI-driven platforms can analyze large datasets to identify risk factors for complications, recommend tailored reconstructive approaches, and facilitate real-time intraoperative assistance through augmented imaging or 3D simulation. These applications have already shown promising results in improving surgical precision and postoperative outcomes [39,40,41].

In the context of autologous fat transfer (AFT), however, the integration of AI remains largely underdeveloped. This gap highlights a significant opportunity for future innovation. AI could potentially assist in selecting ideal donor sites, simulating fat survival scenarios, or standardizing injection protocols to reduce variability in outcomes. Furthermore, machine learning models could support postoperative follow-up by detecting early signs of complications (e.g., fat necrosis or oil cysts) through automated image analysis. As AFT continues to gain popularity, the development of AI-powered tools tailored to its unique biological behavior could lead to more consistent, reproducible, and patient-specific reconstructive strategies.

### 4.1. Study Limitations

This structured narrative review has several limitations. First, although a systematic search was employed using predefined MeSH terms and inclusion/exclusion criteria, the review does not conform to PRISMA guidelines, as it was not designed as a formal systematic review or meta-analysis. As such, certain steps such as risk of bias assessment, protocol registration, and quantitative synthesis were not performed. Second, the analysis is limited by the heterogeneity of the included studies. Differences in study design, grafting techniques, fat processing protocols, outcome definitions (e.g., fat retention, patient satisfaction), and follow-up durations make direct comparisons difficult and reduce the ability to draw firm, generalized conclusions. Third, the decision to exclude irradiated patients allowed for a more homogeneous sample and clearer interpretation of fat graft behavior in non-compromised tissues. However, this approach limits the relevance of the findings to the broader clinical population, where postmastectomy radiation is common and may significantly influence graft survival and complication profiles.

### 4.2. Future Directions

Future research in AFT should aim to optimize graft survival, ensure long-term oncological safety, and promote procedural standardization. The high variability in fat retention—often 30–50% within the first year—highlights the need for innovations such as platelet-rich plasma (PRP), growth factor-enriched scaffolds, and extracellular matrix modulators to improve adipocyte viability and tissue integration.

Standardized, evidence-based protocols are also needed to reduce heterogeneity across studies, particularly in liposuction techniques, fat processing, and injection strategies. Finally, although current data suggest that AFT is oncologically safe, future long-term studies should further investigate the role of ADSC in cancer biology, especially their potential interactions with tumor cells.

Integration of AI-driven personalization into fat grafting could also support shared decision-making by aligning procedural options with individual patient risk profiles and aesthetic goals.

## 5. Conclusions

Autologous fat grafting (AFT) has become a viable technique in breast reconstruction, offering natural aesthetics and high rates of patient satisfaction. While promising, some concerns remain such as fat resorption, procedural variability, and oncological considerations. The integration of artificial intelligence (AI) has significantly improved breast cancer detection and earlier identification of abnormalities in breast imaging. In breast reconstruction, AI is a great tool to predict the fat graft retention, assess potential complication risk, optimize injection techniques offering a more reliable, personalized, and long-lasting solution for patients.

## Figures and Tables

**Table 1:** Comparison of autologous fat transfer (AFT) and implant-based breast reconstruction (IBR).

Study	Publication Year	Type of Study	Key Points
Wederfoort et al. (J Plast Reconstr Aesthet Surg) [18]	2022	Systematic Review	AFT patients had a mean graft survival rate of 37.1%, with ~1/3 of transferred fat remaining viable after 12 months. IBR showed higher volume gain
Piatkowski et al. (JAMA Surg [10]	2023	Randomized Clinical Trial	AFT more expensive initially but cost-effective over 10–30 years. Higher QALY (0.83 vs. 0.79) and BREAST-Q scores in AFT group.
Sungkar et al. (Asian Pac J Cancer Prev) [9]	2024	Meta-Analysis	More IBR patients discontinued due to implant aversion (18) vs. AFT patients due to treatment burden (6).
AlGhanim, K.; et al. (Plast. Surg.) [26]	2025	Systematic Review	IBR results in higher overall satisfaction, while AFT remains a strong alternative for carefully selected patients due to its natural results and fewer implant-related risks
Nguyen et al. (Aesthet Surg J) [27]	2022	Multicenter Observational	AFT had higher complication (3.2% vs. 2.3%) and infection rates (1.1% vs. 0.5%), especially in smokers/high-risk groups.

## Data Availability

No new data were created or analyzed in this study.
